# Phytochemical Profiling and Multitargeted Biological Activities of *Crinum asiaticum* L. var. *anomalum* Baker Leaf: In Vitro and In Silico Insights

**DOI:** 10.3390/plants15131957

**Published:** 2026-06-25

**Authors:** Tue Minh Duong, Son Hoang Nguyen, Kiep Minh Do, Tran Thanh Men, Kenji Kanaori, Kaeko Kamei

**Affiliations:** 1 Department Functional Chemistry, Kyoto Institute of Technology, Kyoto 606-8585, Japan; dmtue2804@gmail.com (T.M.D.);; 2 Faculty of Biochemistry and Food Technology, Vinh Long University of Technology Education, Vinh Long 85000, Vietnam; 3 Faculty of Biology, College of Natural Science, Can Tho University, Can Tho 94000, Vietnam

**Keywords:** *Crinum asiaticum* L. var. *anomalum* Baker, anti-inflammatory, antidiabetic, anticancer, molecular docking, ADMET

## Abstract

This study investigates the phytochemical and pharmacological profiles of *Crinum asiaticum* L. var. *anomalum* Baker from Vietnam. Phytochemical screening identified diverse secondary metabolites, including polyphenols, flavonoids, and alkaloids. Gas chromatography–mass spectrometry analysis of the *n*-hexane fractions revealed 19 major compounds. While all extracts showed moderate antioxidant activity, the chloroform fraction exhibited superior antidiabetic potential via α-amylase inhibition (IC_50_ = 83.13 ± 6.67 µg/mL). Furthermore, at non-cytotoxic concentrations (3.13 to 50 µg/mL), this fraction effectively rescued mouse β-TC6 insulinoma cells from thapsigargin. In anti-inflammatory assays, the *n*-hexane fraction significantly suppressed nitric oxide production in RAW 264.7 macrophages (IC_50_ = 53.12 ± 1.63 µg/mL). Notably, the extracts displayed remarkable selective anticancer activity, particularly the chloroform fraction against HeLa cervical and HepG2/Huh-7 hepatoma cell lines. In silico ADMET and Lipinski’s Rule of Five analyses confirmed that the key bioactive constituents possess favorable pharmacokinetic profiles and drug-likeness. These findings demonstrate *C. asiaticum* L. var. *anomalum* Baker as a promising natural source for developing multitarget therapeutic agents against inflammation, diabetes, and cancer.

## 1. Introduction

The Amaryllidaceae family is globally recognized as a prolific source of bioactive secondary metabolites, with the genus Crinum standing out for its significant pharmacological potential. A defining chemotaxonomic feature of this genus is the presence of distinctive Amaryllidaceae alkaloids, predominantly those containing a crinane-type ring system, such as lycorine. However, despite this shared structural foundation, there is a profound divergence in the chemical profiles among different Crinum species, with each possessing a unique phytochemical map that dictates its specific therapeutic properties [1].

Furthermore, even within a single species, such as *Crinum asiaticum*, significant phytochemical variations occur when plants are cultivated across different geographical regions. While the typical species, *Crinum asiaticum* L., has been extensively characterized in recent bioassays for its baseline phytochemical profile and diverse therapeutic properties [2], its intra-specific divergence is heavily governed by evolutionary genetic adaptation and varying ecological conditions. For instance, phytochemical investigations of *C. asiaticum* var. *sinicum* from China led to the discovery of exclusive, novel alkaloids such as asiaticumines A and B [2]. Moreover, extensive phytochemical profile evaluations have established that the quantitative accumulation and composition of these major bioactive alkaloids vary significantly depending on the specific plant parts utilized—such as leaves, bulbs, or roots—as well as the polarity of the extraction solvents employed [3].

These significant chemical variations have subsequently shaped highly diverse traditional ethnomedicinal applications across different cultures and geographical regions. According to the comprehensive review by Gupta et al., the specialized parts of *C. asiaticum* are distinctively utilized depending on the region; for instance, the leaves are employed to treat rheumatic pain in Bangladesh and fever in Thailand (associated with crinine and phenanthridone derivatives), whereas the alkaloid-rich bulbs and tubers (containing kalbretorine) are favored in India and South Africa for managing tonsillitis and aching joints [4]. Furthermore, the therapeutic utility of this plant widely extends across other countries, including China, Egypt, and the Philippines [5].

Building upon this established principle of geographically and varietally driven chemodiversity, *Crinum asiaticum* L. var. *anomalum* Baker (CAVA), cultivated in Vietnam and locally known as “Ngai to”, represents a highly compelling subject for targeted pharmacological investigation. Morphologically, the *anomalum* variety exhibits unique phenotypic traits, including leaf structural anomalies and altered floral architecture, which distinguish it from the typical, symmetrical *C. asiaticum* species. These macro-botanical distinctions strongly correlate with altered secondary metabolic pathways, yielding distinct chemotypes with specialized pharmacological potencies. Environmental and geographical factors, including the specific tropical microclimates, unique soil conditions, and habitat ecosystems characteristic of the collection regions in Vietnam, play a deterministic role in shaping the plant’s chemical fingerprint. These external ecological stressors directly influence the quantitative and qualitative accumulation of specialized volatile and semi-volatile secondary metabolites within its lipophilic fractions [6]. However, despite its extensive traditional use, scientific validation specifically focusing on the *anomalum* variety remains remarkably scarce. To date, only a single study has investigated its chemical constituents, reporting the first global isolation of three novel substances: (2*R*,3*S*)-7-methoxy-flavan-3-ol, (2*R*,3*S*)-7-hydroxy-flavan-3-ol, and (2*R*,3*S*)-2′-hydroxy-7-methoxy-flavan-3-ol, along with two known flavans, (2*S*)-4′-hydroxy-7-methoxyflavan and (2*S*)-7,4′-dihydroxyflavan from the chloroform leaf extract [7].

Given the complex and interconnected nature of chronic diseases, there is a growing demand to identify natural agents with multitarget therapeutic profiles. Chronic oxidative stress is a well-established precursor that drives inflammatory cascades, malignant cell transformation, and metabolic dysfunction. Therefore, evaluating the antioxidant capacity serves as a fundamental biochemical baseline. Furthermore, managing metabolic disorders, such as diabetes mellitus, requires a comprehensive strategy that simultaneously addresses glycemic control and cellular protection. Chronic hyperglycemia triggers severe oxidative stress through the overproduction of free radicals, which progressively compromises the survival and functionality of pancreatic β-cells. Consequently, identifying natural agents that combine α-amylase inhibitory capacity—to regulate postprandial glucose influx—with robust radical scavenging and cytoprotective properties is essential to shield pancreatic cells from oxidative damage and sustain their endocrine viability [8].

Therefore, the present study was designed to conduct a comprehensive phytochemical screening, including the quantification of total polyphenols, flavonoids, and triterpenoids. Furthermore, we evaluated the biological activities of *Crinum asiaticum* L. var. *anomalum* Baker leaf methanol extract and its solvent fractions, comprehensively focusing on their antioxidant, antidiabetic, anti-inflammatory, and anticancer properties. Additionally, the primary bioactive constituents of the *n*-hexane fractions derived from the leaf methanol extract were identified using a gas chromatography–mass spectrometry (GC-MS) approach. Finally, the pharmacokinetic characteristics and adherence to Lipinski’s Rule of Five for the major identified compounds were assessed using in silico platforms to evaluate their potential as drug candidates.

## 2. Results and Discussion

### 2.1. Phytochemical Composition

Plant-derived secondary metabolites—including steroids, alkaloids, phenolics, and glycosides—exhibit diverse biological properties beneficial to human health, such as anticancer, anti-inflammatory, and antioxidant activities [9,10]. Consequently, phytochemical screening represents a crucial initial step in characterizing the therapeutic potential of medicinal extracts. In this study, the methanol extract of *C. asiaticum* var. *anomalum* leaves was prepared and partitioned into *n*-hexane, chloroform, and ethyl acetate fractions. Initial phytochemical screening via colorimetric methods confirmed the presence of polyphenols, flavonoids, alkaloids, triterpenoids, glycosides, and saponins in both the crude extracts and the resulting solvent fractions (Table 1). However, the ethyl acetate fraction specifically lacked alkaloids, aligning with previous reports on different species of *C. asiaticum* Linn [11]. This observation is likely attributable to the sequential extraction protocol employed; since alkaloids exhibit high solubility in chloroform [12,13], they were presumably exhaustively partitioned into the chloroform fraction during the preceding step. Consequently, the residual alkaloid concentration in the subsequent ethyl acetate fraction likely remained below the detection threshold of the diagnostic reagents.

Polyphenols and flavonoids represent the largest groups of secondary metabolites and are widely distributed in various plant tissues, with significant potential in medicinal chemistry [14,15,16]. Next, the total polyphenol content (TPC) and total flavonoid content (TFC) were determined using the standard curve equations for gallic acid (y = 0.0077x + 0.0283, R^2^ = 0.9999) and quercetin (y = 0.006x − 0.0235, R^2^ = 0.9958), respectively. The total triterpenoid content (TTC) was quantified based on the oleanolic acid (y = 0.0006x + 0.1299, R^2^ = 0.9923) standard. The quantitative distribution is presented in Table 2. Statistical analysis revealed that the ethyl acetate fraction exhibited a significantly higher TPC (111.71 ± 1.63 ^a^ mg GAE/g, *p* < 0.05) compared to all other fractions. Conversely, the methanol extract contained a significantly higher TFC (391.94 ± 5.15 ^a^ mg QE/g) than its sub-fractions. The ethyl acetate fraction exhibited the highest TPC (111.71 ± 1.63 ^a^ mg GAE/g), while the methanol extract showed the highest TFC (391.94 ± 5.15 ^a^ mg QE/g). Notably, the TPC was substantially higher than values previously reported for *C. asiaticum* by Kongkwamcharoen et al. (2021) and Rakhi et al. (2024), which ranged from 12.01 to 52.04 mg GAE/g [17,18]. Similarly, the TFC observed in our extracts surpassed those reported by Goswami (29.8 ± 4.8 to 248.6 ± 6.0 mg QE/g) [19]. These variations in metabolites are widely recognized within the Crinum genus and are typically attributed to intra-specific genetic divergence or variations in the physiological state and growth stages of the plant material [2,3]. In a broader context within the Amaryllidaceae family, it is well-documented that extrinsic environmental parameters, including localized microclimates, soil conditions, altitude, and habitat ecosystems, can significantly shift secondary metabolic pathways. Specifically, these ecological stressors often trigger the upregulation of defensive pathways, directly altering the biosynthesis and accumulation of lipophilic constituents within the plant matrix [1,2,3]. This adaptive metabolic plasticity explains why distinct chemotypes emerge when the same plant lineage adapts to localized geographical regions.

Furthermore, the *n*-hexane fraction possessed the significantly highest TTC (740.17 ± 6.01 ^a^ mg OAE/g, *p* < 0.05), followed by the chloroform fraction (502.94 ± 8.22 ^b^ mg OAE/g). Triterpenoids are essential bioactive components known for their broad biological applications, including anti-inflammatory and anticancer activities [20,21,22]. The high abundance of triterpenoids and flavonoids identified in our extracts likely underpins the diverse pharmacological properties observed in this study. Specifically, these phytochemicals provide a robust basis for the significant antioxidant, antidiabetic, and selective anticancer activities demonstrated in the subsequent section.

### 2.2. In Vitro Antioxidant Activity

Oxidative stress arises from an imbalance between the production of reactive oxygen species (ROS) and the body’s antioxidant defense systems. Uncontrolled free radicals react with vital macromolecules—including proteins, lipids, DNA, and enzymes—leading to metabolic disorders and chronic diseases such as atherosclerosis, cancer, diabetes, and neurodegenerative conditions [23,24]. In this study, the in vitro antioxidant potential of *C. asiaticum* var. *anomalum* extracts and the solvent fractions of the methanol extract were evaluated using DPPH, ABTS+, and reducing power (RP) assays.

**Figure 1:**
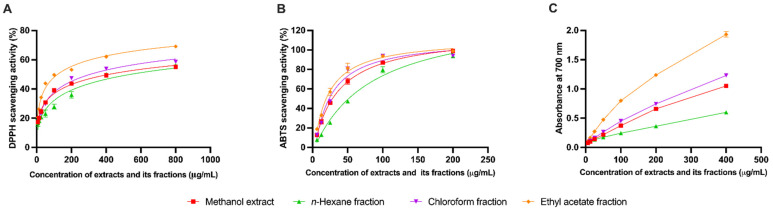
The antioxidant capacity of CAVA leaf methanol extract and the solvent fractions. (**A**) Antioxidant effects evaluated by DPPH radical scavenging activity, (**B**) ABTS+ scavenging activity, and (**C**) reducing power. Data represents the mean ± SD (*n* = 3).

The results, summarized in Figure 1 and Table 3, reveal that the ethyl acetate fraction possesses a significantly stronger antioxidant capacity than the other fractions (*p* < 0.05). It exhibits the significantly lowest IC_50_ value for DPPH (120.00 ± 1.78 ^d^ µg/mL) and the most potent reducing power (Abs_0.5_ = 67.07 ± 2.28 ^d^ µg/mL). For the ABTS assay, the ethyl acetate (18.25 ± 3.65 ^c^ µg/mL) and chloroform fractions (20.41 ± 5.48 ^c^ µg/mL) were significantly more potent than the crude methanol extract and the *n*-hexane fraction, although there was no statistically significant difference between these two top-performing fractions (*p* > 0.05). The superior performance of the ethyl acetate and chloroform fractions correlates strongly with their high TPC and TFC. Phenolic compounds are well-documented for their ability to neutralize free radicals through hydrogen-atom transfer (HAT) or single-electron transfer (SET) mechanisms. Notably, the consistently higher scavenging proficiency observed against ABTS+ compared to DPPH across all fractions can be attributed to the distinct structural and kinetic traits of these two radical species. While the nitrogen-centered DPPH radical is characteristically bulky and exhibits significant steric hindrance that potentially restricts its accessibility to certain structurally complex polyphenols, the planar radical ABTS+ cation is less sterically restricted and operates efficiently through dual SET/HAT pathways, thereby displaying enhanced reactivity toward a broader spectrum of amphiphilic antioxidants present in the extracts. Furthermore, the observed direct correlation between radical scavenging and reducing power aligns with previous reports, suggesting that the mechanism of Fe(III) reduction is chemically analogous to the antioxidant action of phenolics [25].

**Figure 2:**
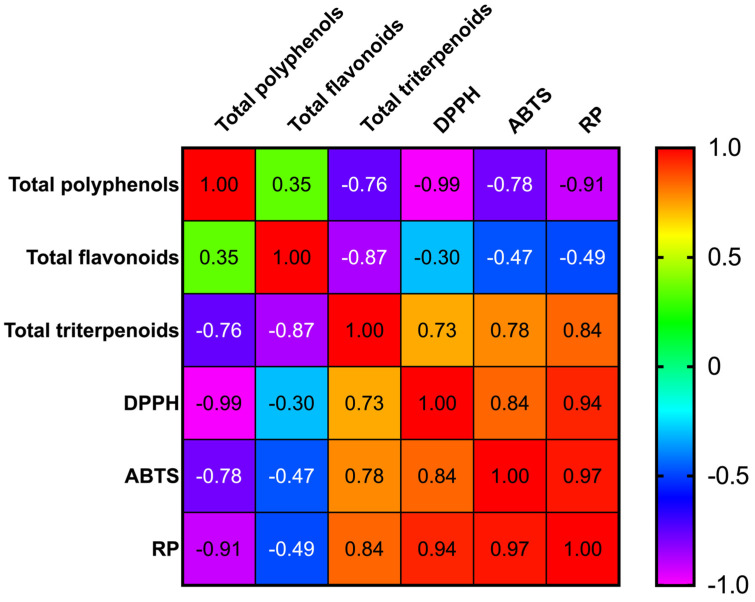
Pearson correlation coefficients between total polyphenols, total flavonoids, total triterpenoids, and antioxidant capacities (the IC_50_ values for DPPH and ABTS+, and Abs_0.5_ values for RP).

To further elucidate these relationships, Pearson correlation coefficients were calculated (Figure 2). The analysis revealed a strong correlation between the TPC/TFC and the antioxidant abilities in the evaluated assays. Specifically, a significant negative correlation was observed between the TPC/TFC and the IC_50_ values of DPPH and ABTS+, as well as the Abs_0.5_ for RP. This indicates that higher TPC and TFC directly contribute to enhanced antioxidant potency (as lower IC_50_ and Abs_0.5_ values signify higher efficiency). In addition, highly robust positive correlations were observed between the respective radical scavenging activities and the iron-reducing power, ranging from very strong (*r* = 0.94 for DPPH) to exceptionally strong (*r* = 0.97 for ABTS+). The high correlation for the ABTS system is consistent with the existing literature [25,26,27] and suggests that the polyphenolic constituents are highly effective in neutralizing radical cations.

Dietary constituents that combine antioxidant and antiglycation properties are essential for modulating metabolic enzymes and physiological processes involved in carbohydrate metabolism. Therefore, the high antioxidant capacity of the ethyl acetate fraction of the leaf methanol extract of *C. asiaticum* L. var. *anomalum* Baker highlights its potential as a therapeutic agent for preventing and managing oxidative-stress-related diseases, including diabetes [28].

### 2.3. In Vitro α-Amylase Inhibitory and β-Cell Cytoprotective Activities of CAVA Leaf Extracts and Solvent Fractions

Diabetes mellitus is a global metabolic disorder characterized by chronic hyperglycemia. While modern pharmacotherapy focuses on glucose regulation, the long-term use of synthetic drugs is often limited by adverse side effects. Consequently, medicinal plants remain essential alternative therapies due to their traditional efficacy and generally lower toxicity [9,29]. In this study, the antidiabetic potential of CAVA leaves was evaluated through α-amylase inhibition and the cytoprotective effects against thapsigargin (TG)-induced cell injury using the mouse β-TC6 insulinoma cell line.

**Figure 3:**
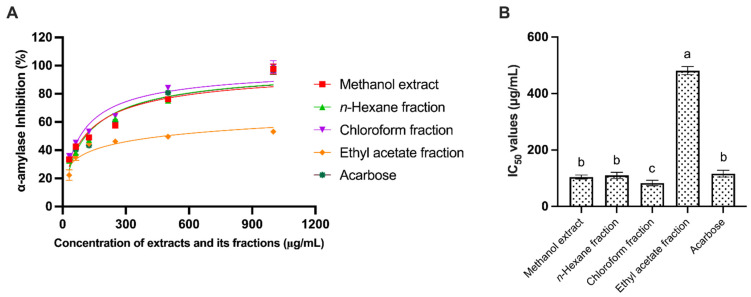
α-Amylase inhibitory activities of CAVA leaf methanol extract and its solvent fractions. (**A**) Dose-dependent α-amylase inhibitory effects of the methanol extract and its sub-fractions; (**B**) IC_50_ values of the methanol extract and its sub-fractions. Acarbose was used as a positive control. The results are presented as the mean ± SD (*n* = 3). Different superscript letters (a, b and c) within the same column indicate statistically significant differences among groups based on One-way ANOVA followed by Tukey’s multiple comparison test (*p* < 0.05).

The α-amylase inhibitory activities are presented in Figure 3. Statistical analysis indicated that the chloroform fraction exhibited a significantly stronger inhibitory effect (IC_50_ = 83.13 ± 6.67 ^c^ µg/mL, *p* < 0.05) compared to the positive control, acarbose (IC_50_ = 116.3 ± 2.11 ^b^ µg/mL). The methanol extract (104.32 ± 7.42 ^b^ µg/mL) and the *n*-hexane fraction (110.10 ± 7.09 ^b^ µg/mL) showed comparable activity to acarbose, with no statistically significant differences observed among these three groups (*p* > 0.05). The remaining fractions demonstrated significantly weaker inhibition. Interestingly, our results revealed that the ethyl acetate fraction, despite harvesting the highest abundance of total phenolic compounds, displayed the weakest inhibitory efficacy against α-amylase. This counter-intuitive phenomenon underscores that crude quantitative phenolic content does not strictly parallel targeted enzymatic inhibition. Instead, the outcome is heavily governed by internal structure–activity dynamics and inter-molecular behaviors. On one hand, the highly concentrated and complex mixture of phenolics within the ethyl acetate matrix may induce profound antagonistic effects or steric hindrance, effectively blocking individual molecules from navigating into the catalytic pockets of the enzyme [30]. On the other hand, the superior inhibitory potencies observed in less polar fractions (such as chloroform and *n*-hexane) strongly suggest the presence of specific, highly complementary non-polar or semi-polar scaffolds—such as distinctive triterpenoids, alkaloids, or lipophilic volatile compounds identified via GC-MS (detailed in Section 2.5)—that exhibit superior binding configurations and thermodynamic stability within the α-amylase active site. It is worth noting that while organic solvents are employed for fractionation, the practical value of these tests lies in the identification of potential lead compounds. By segregating complex phytochemical mixtures into fractions based on polarity, we can isolate and characterize specific bioactive constituents—which might otherwise remain masked in crude aqueous preparations. These identified lipophilic or semi-polar scaffolds provide the necessary chemical blueprints for the future development of standardized therapeutic formulations. Specifically, this approach facilitates the design of advanced delivery systems (such as lipid-based carriers or nano-encapsulation) [31] to ensure that such bioactive phytoconstituents—which are inherently responsible for the observed α-amylase inhibition—can effectively reach their target sites within the human gastrointestinal tract upon oral administration. These empirical findings suggest that the active principles of *C. asiaticum* var. *anomalum* are predominantly concentrated within the low-to-medium polarity matrices, effectively suppressing starch hydrolysis and thereby offering a valuable biochemical foundation for mitigating postprandial blood glucose spikes.

**Figure 4:**
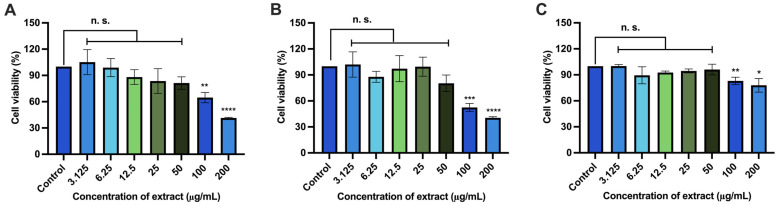
Effects of CAVA leaf methanol extract and its solvent fractions on the cell viability of β-TC6 cells. β-TC6 cells were treated with the indicated samples for 24 h, followed by cell viability assessment. (**A**) Effect of the methanol extract, (**B**) effect of the *n*-hexane fraction, and (**C**) effect of the chloroform fraction on cell viability. Results are presented as the mean ± SD (*n* = 3). n. s., not significant. * *p* < 0.05; ** *p* < 0.01; *** *p* < 0.001, **** *p* < 0.0001 vs. the control group.

Based on their potent enzyme inhibitory activities, the methanol extract and its chloroform and *n*-hexane fractions were selected for further evaluation using β-TC6 cells. Initial cell viability assays (Figure 4) revealed that at 200 µg/mL, the methanol extract and *n*-hexane fraction significantly reduced cell viability to 40.43% and 41.41%, respectively. In contrast, the chloroform fraction maintained a high survival rate of 84.56% at the same concentration. Since the methanol extract and all sub-fractions exhibited negligible toxicity at concentrations of 50 µg/mL and below, this selected range was employed in subsequent experiments to investigate their potential cytoprotective effects against cellular damage induced by TG.

**Figure 5:**
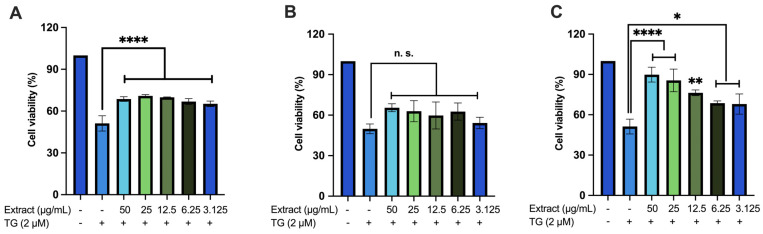
Protective effects of CAVA leaf methanol extract and its solvent fractions against TG-induced injury in β-TC6 cells. β-TC6 cells were pre-treated with the methanol extract, *n*-hexane fraction, or chloroform fraction for 24 h, followed by co-treatment with the samples and/or 2 μM TG for an additional 24 h. (**A**) Protective effect of the methanol extract, (**B**) protective effect of the *n*-hexane fraction, and (**C**) Protective effect of the chloroform fraction. Results are presented as mean ± SD (*n* = 3). n. s., not significant. * *p* < 0.05; ** *p* < 0.01; **** *p* < 0.0001 vs. the TG-treated control group.

The results of the cytoprotective assay against thapsigargin-induced injury are presented in Figure 5. Thapsigargin is a potent inducer of cellular stress and cytotoxicity, which serves as a key pathological model contributing to β-cell failure in diabetes. Our findings demonstrated that the *n*-hexane fraction exhibited a slight upward trend in cell viability, although this protective tendency did not reach statistical significance (*p* > 0.05) compared to the TG-treated group. Notably, the chloroform fraction exhibited superior cytoprotective efficacy, maintaining over 80% cell viability at 50 µg/mL, whereas the methanol extract provided only moderate protection (approximately 60%). Even at the lowest concentration of 3.13 µg/mL, the methanol extract and its chloroform fraction sustained statistically significant protective effects. These results suggest that the bioactive compounds concentrated within these specific matrices may effectively modulate cellular stress responses or alleviate oxidative stress, thereby preserving insulin-producing cells under metabolic stress. Mechanistically, this superior cytoprotective effect can be strongly correlated with the high quantitative abundance of total triterpenoids (TTC) in both the *n*-hexane and chloroform fractions, alongside the selective retention of alkaloids within the chloroform matrix (Table 1 and Table 2). Given their highly lipophilic nature, plant-derived triterpenoids and alkaloids can readily integrate into cellular membranes, where they help stabilize the lipid bilayer against chemically induced structural damage. Furthermore, these secondary metabolites are widely documented to neutralize intracellular reactive intermediates, preserve mitochondrial integrity, and suppress pro-apoptotic cascades under severe toxic insults. Therefore, the enrichment of these specialized phytochemicals in the chloroform fraction likely forms a robust synergistic shield, effectively counteracting the cellular injury induced by thapsigargin and maintaining the overall survival of the pancreatic β-cells. Although thapsigargin was utilized as a recognized model for inducing severe cellular injury, our findings at this stage focus strictly on the preservation of cell viability (cytoprotection) rather than the direct modulation of specific intracellular organelle stress signaling pathways. Consequently, while these data demonstrate significant pharmacological potential, further mechanistic investigations are essential to fully elucidate the precise molecular pathways involved.

### 2.4. Anticancer Potential of CAVA Leaf Extracts

The dose-dependent anticancer potential of CAVA leaf extracts was evaluated against various human cancer cell lines using the CCK-8 assay. A critical parameter in anticancer drug discovery is the selectivity index (SI), which is defined as the ratio of the IC_50_ value for normal human embryonic kidney cells (HEK293) to that for cancer cells. A higher SI indicates that the extract exhibits greater selective toxicity toward malignant cells while minimizing adverse effects on healthy tissues.

**Figure 6:**
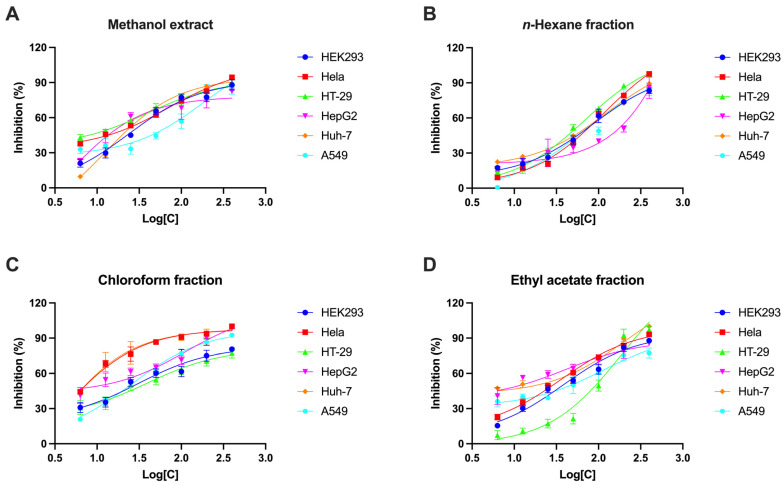
Effects of CAVA leaf extracts and solvent fractions prepared from the methanol extract on the cell viability of cancer cells and normal cells. HEK293 (non-cancerous control) and cancer cells were treated with increasing concentrations of the methanol extract or the *n*-hexane, chloroform, and ethyl acetate fractions for 48 h. Cell viability was subsequently assessed using the CCK-8 assay. Graphs represent: (**A**) methanol extract, (**B**) *n*-hexane fraction, (**C**) chloroform fraction, and (**D**) ethyl acetate fraction. Data are presented as mean ± SD (*n* = 3).

The cytotoxicity of CAVA extracts and their respective fractions was evaluated against both normal (HEK293) and various cancer cell lines. As illustrated in Figure 6, all tested samples exhibited dose-dependent inhibitory effects on cell viability. The IC_50_ values for the methanol extract, as well as the *n*-hexane, chloroform, and ethyl acetate fractions (derived from the methanol extract) against HEK293 cells, were 23.21 ± 4.20, 87.70 ± 3.95, 30.81 ± 4.98 and 37.65 ± 4.78 µg/mL, respectively. Notably, the *n*-hexane fraction (Figure 6B) showed the highest IC_50_ value for the HEK293 cells, indicating the lowest toxicity toward normal cells among all preparations.

The IC_50_ and SI values for the cervical (HeLa), colorectal (HT-29), liver (Huh-7, HepG2), and lung (A549) cancer cell lines are summarized in Table 4. The extracts demonstrated significant cytotoxicity, with the chloroform fraction exhibiting the most potent activity, characterized by IC_50_ values ranging from 7.10 to 33.21 µg/mL. According to the United States National Cancer Institute (NCI) criteria, crude botanical extracts are considered promising candidates for further purification if they exhibit IC_50_ values lower than 20 µg/mL or 30 µg/mL after 48–72 h of incubation [32,33]. Nevertheless, it has been suggested that extracts may still be considered effective candidates at concentrations up to 100 µg/mL [34,35]. Notably, the chloroform fraction fulfilled these stringent criteria across all tested cell lines, supporting its potential for further bioassay-guided fractionation. Beyond potency, an ideal anticancer agent should possess an SI value ≥ 2. Based on these combined criteria, the chloroform fraction emerged as the superior candidate, showing strong and selective toxicity against HeLa (IC_50_ = 7.10 µg/mL, SI = 4.34), Huh-7 (IC_50_ = 7.24 µg/mL, SI = 4.26), and HepG2 (IC_50_ = 10.69 µg/mL, SI = 2.88). Additionally, the methanol extract showed selective toxicity toward HT-29 cells (SI = 2.14), while the ethyl acetate fraction targeted liver cancer lines (Huh-7 and HepG2) with SI values > 2.5. This pronounced biological activity is fundamentally driven by the distinctive phytochemical distribution across the solvent matrices. Specifically, the exceptional cytotoxic potency and high selectivity indices of the chloroform fraction (SI = 4.34 against HeLa and 4.26 against Huh-7) can be directly linked to its unique co-enrichment of both alkaloids and a high concentration of triterpenoids (502.94 mg OAE/g), as detailed in Table 1 and Table 2. Plant alkaloids and triterpenoids are well-established to exert profound anti-proliferative activities by disrupting cancer cell membrane frameworks, inducing cell cycle arrest, and triggering apoptotic cascades through caspase activation. The co-existence of these highly active scaffolds within the chloroform fraction likely induces a powerful synergistic effect, allowing it to aggressively target malignant cells while minimizing toxicity toward healthy embryonic kidney cells (HEK293). In contrast, despite possessing the highest polyphenol content (TPC), the ethyl acetate fraction displayed lower or more restricted cytotoxicity against certain lines, which can be attributed to the complete absence of alkaloids in its matrix. This structural bifurcation clearly demonstrates that crude quantitative phenolic abundance alone does not dictate anticancer efficacy; rather, the structural diversity and the presence of specialized lipophilic metabolites, such as alkaloids and triterpenoids, are the critical determinants of selective tumor cell destruction. Notably, while the chloroform fraction demonstrated the most aggressive cytotoxic profile, the *n*-hexane fraction presented the lowest toxicity toward healthy cells (IC_50_ = 87.70 ± 3.95) µg/mL on HEK293) along with moderate selectivity toward HT-29 and Huh-7 lines. This favorable safety window justified further pharmacological evaluation of the *n*-hexane fraction in subsequent comparative assays.

### 2.5. Anti-Inflammatory Effects of CAVA Leaf Extracts and Molecular Docking Analysis

RAW 264.7 macrophages are a well-established model for evaluating the anti-inflammatory activity of natural products [36,37]. Upon stimulation with lipopolysaccharide (LPS), these cells initiate a robust inflammatory response characterized by the overproduction of key mediators, including nitric oxide (NO) and pro-inflammatory cytokines. In the present study, we evaluated the anti-inflammatory potential of *C. asiaticum* var. *anomalum* leaf extracts and solvent fractions by assessing their ability to modulate these inflammatory markers.

**Figure 7:**
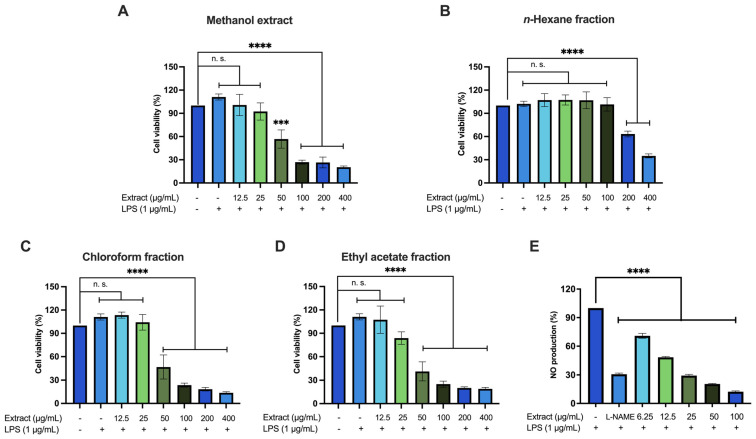
Effects of CAVA leaf extracts and solvent fractions on the cell viability and NO production in RAW 264.7 macrophages. To assess cytotoxicity, cells were treated with various concentrations of the methanol extracts or solvent fractions derived from the methanol extract for 24 h, after which cell viability was determined. Cell viability results are shown for (**A**) the methanol extract, (**B**) *n*-hexane fraction, (**C**) ethyl acetate fraction, and (**D**) chloroform fraction. (**E**) To evaluate the anti-inflammatory activity of the *n*-hexane fraction, cells were pre-treated with the fraction for 24 h, followed by co-treatment with LPS for an additional 24 h. Subsequently, NO levels in the culture medium were quantified. Results are presented as the mean ± SD (*n* = 3). n. s., not significant. *** *p* < 0.001, and **** *p* < 0.0001 vs. the control group.

The cytotoxicity of the extracts on RAW 264.7 cells was initially assessed using the CCK-8 assay (Figure 7A–D). At a concentration of 400 µg/mL, the methanol extract, as well as the chloroform and ethyl acetate fractions derived from the methanol extract, exhibited significant cytotoxicity. Notably, the *n*-hexane fraction displayed negligible toxicity at 100 µg/mL, while the other fractions maintained cell viability only at concentrations below 25 µg/mL. The selection of the *n*-hexane fraction for subsequent anti-inflammatory evaluation (NO assay, GC-MS, and in silico docking) was driven by a dual chemical and biological rationale. From a biological perspective, in evaluating NO inhibition, it is crucial to ensure that the reduction in NO levels is a genuine pharmacological effect (i.e., enzyme inhibition) rather than a false positive caused by a decline in cell viability. The *n*-hexane fraction exhibited the widest therapeutic window (negligible toxicity up to 100 µg/mL), providing a robust platform to assess true anti-inflammatory mechanisms. From a chemical perspective, as previously established in Table 3, this fraction is exceptionally rich in triterpenoids (740.17 mg OAE/g)—a class of lipophilic metabolites universally recognized for their potent anti-inflammatory properties. While it is highly plausible that other fractions (such as chloroform or ethyl acetate) harbor highly potent anti-inflammatory constituents active at lower, sub-toxic concentrations, the optimal combination of high triterpenoid abundance and excellent macrophage tolerability made the *n*-hexane fraction the most compelling candidate for immediate lead-compound identification. Based on these findings, the *n*-hexane fraction was selected for further investigation of its inhibitory effects on NO production in LPS-stimulated cells. As shown in Figure 7E, the *n*-hexane fraction suppressed NO production in a dose-dependent manner, exhibiting an IC_50_ value of 50 µg/mL. To accurately benchmark this pharmacological efficacy, L-NAME (*N*-Nitro-*L*-arginine methyl ester), a standard NOS inhibitor, was employed as a positive control. At a concentration of 100 µM, L-NAME reduced NO production to 30.68%. These results suggest that the non-polar constituents of this variety possess significant anti-inflammatory potential. Furthermore, the resulting freeze-dried fractions demonstrated a favorable safety profile in our cell viability assays, indicating that after the thorough removal of organic solvents, these bioactive scaffolds are well-tolerated by the tested cell lines.

**Figure 8:**
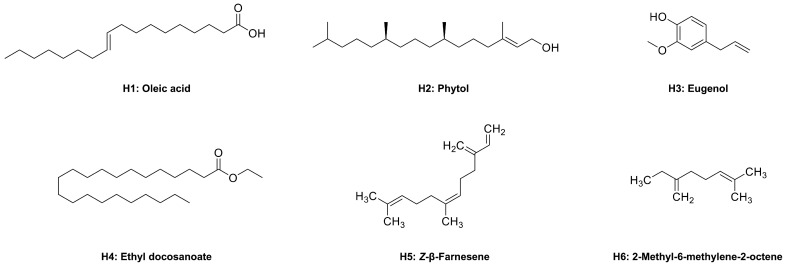
Main compounds of the *n*-hexane fraction.

This potent anti-inflammatory efficiency of the *n*-hexane fraction strongly substantiates the quantitative findings in Table 2, where the *n*-hexane matrix exhibited the absolute highest accumulation of total triterpenoids (740.17 mg OAE/g). Triterpenoids are class-leading lipophilic inhibitors of inflammatory cascades, known to suppress the activation of nuclear factor-kappa B (NF-κB) and downregulate downstream inflammatory enzymes. Phytochemical compounds within the *n*-hexane fraction were analyzed using GC-MS (Figure S1), leading to the identification of 19 major compounds (Table A1). The chemical structures of predominant constituents are shown in Figure 8, including oleic acid (H1, 82.51%), phytol (H2, 5.78%), eugenol (H3, 2.33%), ethyl docosanoate (H4, 1.59%), (*Z)*-β-farnesene (H5, 1.47%), and 2-methyl-6-methylene-2-octene (H6, 1.30%), with all values expressed as the relative peak area percentage of each compound to the total peak area of the identified predominant constituents. The high abundance of oleic acid (82.51%) and phytol (5.78%) in the *n*-hexane fraction was fully anticipated, as these hydrophobic molecules align with the non-polar nature of the solvent. Such lipophilic profiles are highly characteristic of *n*-hexane extracts from *Crinum* species. To bridge the gap between these chemical findings and the observed biological effects, molecular docking simulations were performed. The primary components identified by GC-MS were docked against two key inflammatory enzymes: inducible nitric oxide synthase (iNOS) and cyclooxygenase-2 (COX-2). This in silico approach aimed to identify specific metabolites from the GC-MS profile responsible for the enzyme inhibition. Using PyRx 0.8, the binding sites were defined based on the coordinates of co-crystallized ligands within the respective target proteins. To establish a rigorous mechanistic baseline, molecular docking was executed using celecoxib (a clinically established selective COX-2 inhibitor) and indomethacin (a classic non-selective pan-COX inhibitor) as positive controls. The resulting binding affinities are presented in Table S1. Celecoxib exhibited powerful binding energies of −10.0 kcal/mol for iNOS and −11.40 kcal/mol for COX-2, with its superior affinity toward COX-2 structurally reflecting its well-documented selectivity. Meanwhile, indomethacin demonstrated robust binding affinities of −8.90 kcal/mol for iNOS and −7.40 kcal/mol for COX-2. The binding affinities of the identified phytochemical constituents ranged from –6.1 to −7.4 kcal/mol for iNOS and from −5.80 to −7.20 kcal/mol for COX-2. Notably, phytol—one of the major constituents of the *n*-hexane fraction—exhibited the highest binding affinity among the tested compounds, with values of −7.40 kcal/mol for iNOS and −7.20 kcal/mol for COX-2. While these values are lower than the optimized synthetic profile of celecoxib, the close proximity of phytol’s binding energies to those of indomethacin indicates a highly spontaneous, thermodynamically stable interaction. This structural alignment suggests that phytol can effectively occupy the hydrophobic catalytic domains of both iNOS and COX-2, successfully rationalizing the in vitro anti-inflammatory and cytoprotective properties observed for the *n*-hexane fraction.

**Figure 9:**
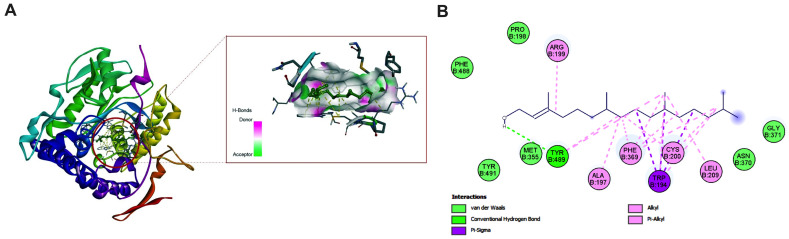
Molecular docking of phytol within the active site of the iNOS enzyme (PDB: 4NOS). (**A**) 3D representation showing the ligand-binding pocket and spatial orientation; (**B**) 2D interaction diagram illustrating specific chemical bonds and amino acid residues involved in the complex stabilization.

**Figure 10:**
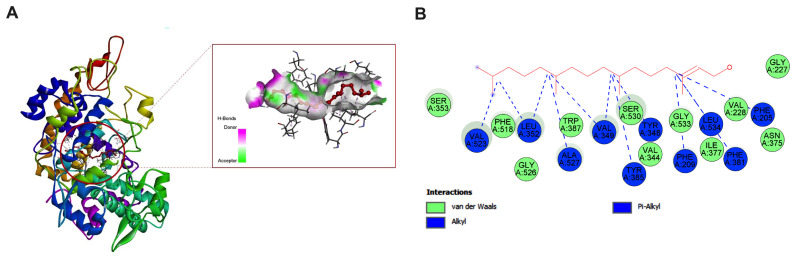
Molecular docking of phytol within the active site of the COX2 enzyme (PDB: 5KIR). (**A**) 3D representation showing the ligand-binding pocket and spatial orientation; (**B**) 2D interaction diagram illustrating specific chemical bonds and amino acid residues involved in the complex stabilization.

Detailed interaction analysis revealed that phytol is stabilized within the iNOS binding pocket through van der Waals interactions with residues Pro198, Phe488, Tyr491, Met355, Asn370, and Gly371. Furthermore, it forms a network of hydrogen bonds, alkyl interactions, and π-alkyl bonds with residues such as Arg199, Tyr489, Ala197, Phe369, Cys200, and Leu209 (Figure 9). Similarly, the stability of the phytol/COX-2 complex is maintained by van der Waals forces with Gly227, Asn375, Val223, Ile377, Gly533, Ser530, Trp387, Gly525, Phe518, and Ser353, alongside alkyl and hydrogen bonds with key residues including Val523, Leu352, Ala527, Val349, Tyr385, Tyr348, Phe209, Leu534, Phe381, and Phe205 (Figure 10). The potent binding affinities observed for the identified metabolites are intrinsically linked to their specific structural motifs and spatial topographies. Phytol, a branched-chain diterpene alcohol, possesses a unique amphiphilic character. Its extended hydrophobic tail fits precisely into the deep lipophilic channels of both iNOS and COX-2, maximizing localized van der Waals, hydrophobic, and alkyl-type interactions with conserved hydrophobic residues (such as Leu, Val, and Phe). Concurrently, the terminal hydroxyl group of phytol serves as a directional hydrogen-bond donor/acceptor, providing a critical electrostatic anchor to polar residues within or adjacent to the catalytic domains. Similarly, the polar carboxyl moiety of long-chain fatty acids and the oxygenated functional groups (methoxy and hydroxyl substituents) of aromatic and carbohydrate derivatives (e.g., eugenol and modified saccharides) offer multiple geometrically favorable sites for directional hydrogen bonding. The clear structural synergy between these rigid or flexible lipophilic backbones and polar functional moieties effectively locks the ligands within the enzymatic active sites. This multitargeted steric blockade likely hinders the spatial entry of natural substrates, thereby molecularly rationalizing the observed enzymatic and cellular inhibition.

In summary, the integration of in vitro experiments and in silico molecular docking suggests that CAVA leaf extracts—particularly the *n*-hexane fraction and its major constituent phytol—could serve as effective, low-toxicity candidates for the development of anti-inflammatory therapeutics. Given that chronic inflammation is a key precursor to severe conditions, such as cancer and neurological disorders, the discovery of such plant-derived agents holds significant therapeutic potential for future drug discovery and preventive medicine.

### 2.6. ADMET and Drug-Likeness Prediction of Bioactive Compounds in the n-Hexane Fraction

Pharmacokinetics—comprising absorption, distribution, metabolism, and excretion (ADME)—characterizes the interaction between the physiological environment and medicinal compounds [38,39]. A comprehensive understanding of these parameters is essential for optimizing therapeutic efficacy and safety. Given the potent in vitro antidiabetic and anti-inflammatory activities demonstrated by the *n*-hexane fraction, its primary constituents were evaluated for their pharmacokinetic profiles and drug-likeness.

Lipinski’s “Rule of Five” serves as a widely recognized framework for assessing the drug-likeness and oral bioavailability of chemical compounds. According to this rule, a molecule is generally classified as drug-like if it fulfills at least three of the four criteria (with no more than one violation): (1) molecular weight ≤ 500 Da, (2) lipophilicity (log *P* ≤ 5), (3) fewer than 5 hydrogen bond donors, and (4) fewer than 10 hydrogen bond acceptors [40]. Additionally, molar refractivity (MR) between 40 and 130 is frequently employed as a supplementary parameter for drug-likeness. In this study, we evaluated the six major constituents (H1–H6) identified in the *n*-hexane fraction, using the SwissADME tool (https://www.swissadme.ch accessed on 14 November 2025). The results indicated that all compounds exhibited favorable drug-like profiles. Specifically, while compounds H3 and H6 strictly adhered to all requirements, all other compounds satisfied four of the five evaluated criteria (Table 5).

**Figure 11:**
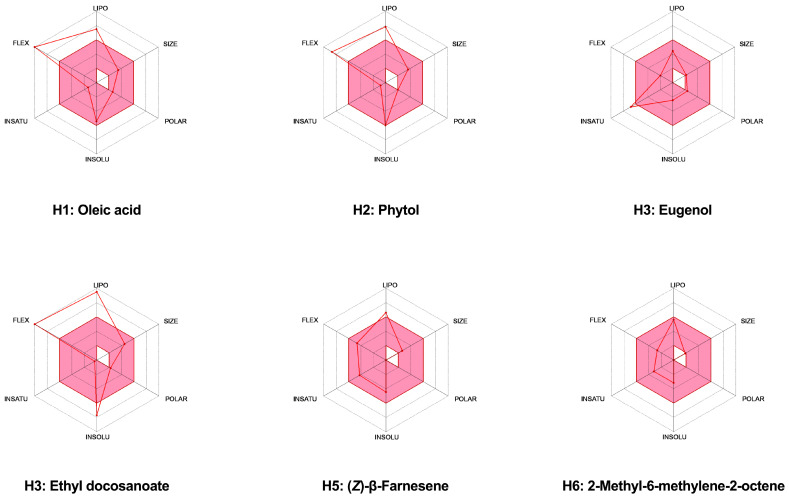
Bioavailability radar charts of the major compounds identified in the *n*-hexane fraction. The evaluated parameters include molecular weight (SIZE), polarity (POLAR), insolubility (INSOLO), unsaturation (INSATU), conformational flexibility (FLEX), and lipophilicity (LIPO). The red line represents the values for each assessed compound, while the pink-shaded area indicates the optimal range for oral bioavailability.

The physicochemical properties were further visualized using the SwissADME bioavailability radar (Figure 11), which assesses six key characteristics: lipophilicity, size, polarity, solubility, saturation, and conformational flexibility. A comparative topological analysis of the radar charts revealed a clear structural bifurcation among the evaluated phytoconstituents. The radar plots showed that the descriptors for the compounds of H3, H5, and H6 were localized within the optimal, pink-shaded region. This strict alignment strongly predicts high gastrointestinal absorption and favorable theoretical oral bioavailability for these low-molecular-weight terpenoid and phenylpropanoid derivatives. A distinct exception was observed for the compounds of H1, H2, and H4, which displayed elevated lipophilicity, placing them outside the ideal range for these specific criteria. In particular, high FLEX (Flexibility) values can lead to a decrease in the bioavailability of these compounds.

The ADMET and pharmacokinetic profiles of the identified compounds (H1–H6) from the *n*-hexane fraction are summarized in Table 6. Regarding biopharmaceutical absorption, the evaluated phytoconstituents exhibited a predominantly higher lipophilicity over water solubility, driven by predicted solubility values well below the standard threshold of −4.0 log mol/L. This structural lipophilicity directly accounts for their exceptional predicted human intestinal absorption percentages, which are tightly clustered within a high-efficacy range of 89.854% to 94.917%, paired with optimal Caco-2 permeability values that uniformly surpass the high-permeability benchmark of 0.90 log *P* in 10^−6^ cm/s. This uniform high-permeability profile is consistent with the structure–activity relationship (SAR) of non-polar matrices, where the absolute dominance of long-chain fatty acids, hydrophobic esters, and terpenoid skeletons facilitates seamless passive transcellular transport across epithelial lipid bilayers. Evaluating dermatological parameters provides additional insights into the topical development potential of this matrix, where compounds H3, H5 and H6 safely surpassed the standard high-penetration skin permeability threshold (logKp > −2.5), suggesting that they can act as potent natural penetration enhancers by temporarily disrupting the highly organized lipid architecture of the stratum corneum [41].

For systemic distribution, the steady-state volume of distribution (VDss) remained below the high tissue-partitioning threshold of 0.45 L/kg for the major constituents except H5, suggesting a localized distribution within the plasma matrix. Furthermore, due to their compliant lipid-soluble architectures, most components demonstrated strong central nervous system accessibility by crossing the blood–brain barrier readily (logBB > 0.3), with the notable exception of H1, which exhibited restricted blood–brain barrier permeability.

From a metabolic standpoint, the entire molecular cohort exhibited general metabolic neutrality, as none of the compounds acted as substrates or inhibitors for the critical metabolic enzyme CYP2D6, minimizing the risk of drug–drug interactions or slowed breakdown of co-administered therapeutic substances. Total clearance values reflected a well-regulated physiological turnover, where H1, H2, H4 and H5 displayed elevated elimination rates to prevent toxic tissue accumulation, whereas the lower clearance profiles of H3 and H6 predict a more prolonged systemic half-life. Crucially, toxicological screening yielded highly promising safety profiles that strongly support the low in vitro cytotoxicity previously observed on normal HEK293 cells, as the evaluated constituents were generally devoid of Ames mutagenicity and showed a complete absence of hepatotoxicity or significant organ-specific toxicity towards the heart. Although certain compounds like H2 and H4 flagged potential cardiotoxicity trends in high-dose computational models, their overall compliant profiles support a high therapeutic safety margin. Regarding dermal safety, the concentrated lipophilic structures (H1–H5) were flagged as potential skin sensitisers, a trait frequently observed with active volatile terpenes and free fatty acids when interacting with epidermal lipid frameworks; this indicates that topical deployment should be carefully optimized within controlled, diluted formulations to mitigate localized allergic dermatitis while maximizing the transdermal efficacy established under the same biophysical transport mechanisms [42].

These individual pharmacokinetic trajectories align seamlessly with established global health applications and chemical synergies, where oleic acid (H1) is globally recognized by the World Health Organization (WHO) and Food and Agriculture Organization of the United Nations as a beneficial monounsaturated fatty acid that suppresses chronic low-grade inflammation and protects against metabolic disorders [43,44] and phytol (H2) is widely documented for its anti-proliferative, antioxidant, and multitarget anti-inflammatory properties via the down-regulation of pro-inflammatory cytokines [45,46]. Furthermore, eugenol (H3) represents a landmark natural molecule traditionally utilized in pharmaceutical applications for its profound localized anesthetic and anti-inflammatory properties with a long-standing therapeutic use fully accepted by the WHO [47,48], while the volatile sesquiterpene (*Z*)-β-farnesene (H5) and the aliphatic hydrocarbon 2-methyl-6-methylene-2-octene (H6) contribute significantly to the overall biological synergy through their intense membrane-stabilizing and radical scavenging capabilities [49], complemented by ethyl docosanoate (H4) which reinforces the lipophilic matrix to enhance compound stability and cellular membrane fusion. In conclusion, the excellent absorption kinetics, low predicted organ toxicity, and established global health profiles provide a robust pharmacokinetic validation for the *n*-hexane fraction as a safe, active therapeutic matrix.

**Table 6:** ADMET profiles of the six major compounds (H1–H6) identified in the *n*-hexane fraction.

Property	Model Name	Units	H1	H2	H3	H4	H5	H6
Absorption	Water solubility	log mol/L	−5.924	−7.535	−2.25	−7.869	−6.956	−4.64
	Caco-2 permeability	log *P* in 10^–6^ cm/s	1.563	1.399	1.559	1.091	1.405	1.405
	Intestinal absorption (human)	% absorbed	91.823	90.643	92.041	89.854	93.432	94.917
	Skin permeability	log K*p*	−2.725	−2.631	−2.207	−2.819	−1.281	−1.038
Distribution	VDss	log L/kg	−0.558	0.385	0.24	0.238	0.574	0.375
	BBB permeability	log BB	−0.168	0.793	0.374	0.872	0.835	0.776
Metabolism	CYP2D6 substrate	Yes/No	No	No	No	No	No	No
	CYP3A4 substrate	Yes/No	Yes	Yes	No	Yes	No	No
	CYP1A2 inhibitor	Yes/No	Yes	Yes	Yes	Yes	No	No
	CYP2C19 inhibitor	Yes/No	No	No	No	No	No	No
	CYP2C9 inhibitor	Yes/No	No	No	No	No	No	No
	CYP2D6 inhibitor	Yes/No	No	No	No	No	No	No
	CYP3A4 inhibitor	Yes/No	No	No	No	No	No	No
Excretion	Total clearance	log mL/min/kg	1.884	1.686	0.282	2.112	1.832	0.436
Toxicity	Ames toxicity	Yes/No	No	No	Yes	No	No	No
	hERG I inhibitor	Yes/No	No	No	No	No	No	No
	hERG II inhibitor	Yes/No	No	Yes	No	Yes	No	No
	Hepatotoxicity	Yes/No	No	No	No	No	No	No
	Skin sensitisation	Yes/No	Yes	Yes	Yes	Yes	Yes	No

H1: oleic acid; H2: phytol; H3: eugenol; H4: ethyl docosanoate; H5: (*Z*)-β-farnesene; H6: 2-methyl-6-methylene-2-octene. Caco-2 permeability > 0.9; Intestinal absorption (human) 30–50%; Skin permeability > −2.5; V_Dss_ (human) > 0.45; BBB permeability > 0.3; Total clearance: higher is better [50].

## 3. Materials and Methods

### 3.1. Materials

The leaves of *Crinum asiaticum* L. var. *anomalum* Baker were collected in Vietnam. This material belongs to the same batch as the sample utilized in a previous investigation [7]. The botanical identity was verified by Associate Professor Dr. Tran Thanh Men (Department of Biology, Faculty of Natural Sciences, Can Tho University) and Phung Thi Hang (Faculty of Biology Education, Can Tho University) according to the Vietnamese plant taxonomy system. A voucher specimen (Voucher No: CriAsi.NHS-002) has been deposited at the plant laboratory of the Department of Biology, Can Tho University, Vietnam. Unless otherwise stated, all chemicals and reagents were obtained from FUJIFILM Wako Pure Chemical Corp. (Tokyo, Japan). Cell Counting Kit-8 (CCK-8) was obtained from Dojindo Laboratories (Kumamoto, Japan).

Cell lines were obtained from the Japanese Collection of Research Bioresources (JCRB) Cell Bank (Osaka, Japan), including HT-29 colorectal cancer cells (No. JCRB1383), HepG2 liver cancer cells (No. JCRB1592), and Huh-7 liver cancer cells (No. JCRB0403), A549 lung cancer cells (No. JCRB0076), HeLa cervical cancer cells (No. JCRB9004), and HEK293 normal kidney cells (No. JCRB9068). Additionally, β-TC6 mouse pancreatic cells (No. CRL-3605) and RAW 264.7 murine leukemia macrophage cells (No. TIB-71) were obtained from the American Type Culture Collection (ATCC, Manassas, VA, USA). The cells were cultured in Dulbecco’s Modified Eagle Medium (DMEM; Fujifilm, Tokyo, Japan) or Eagle’s Minimum Essential Medium (EMEM; Nacalai Tesque, Kyoto, Japan). All media were supplemented with 10% fetal bovine serum (FBS, Biosera, Nuaille, France) and 1% penicillin/streptomycin (Fujifilm, Tokyo, Japan). The cells were maintained in a humidified atmosphere containing 5% CO_2_ at 37 °C.

### 3.2. Preparation of Extracts and Solvent Fractions

To prepare the methanolic extract, 150 g of dried powdered leaves were sonicated with 4 L of methanol for 90 min at 30 °C [7]. This process was repeated four times, and the combined filtrates were concentrated under reduced pressure to yield 30.30 g of the methanol extract. The solvent fractions were initially concentrated under reduced pressure using a rotary evaporator (Advantec, Tokyo, Japan) and then lyophilized (freeze-dried) to obtain dry extracts, thereby ensuring the complete removal of any residual solvents. Subsequently, a portion of this crude extract (28.50 g) was suspended in 300 mL of distilled water. This aqueous suspension was then subjected to sequential liquid–liquid partitioning using solvents of increasing polarity, resulting in the following fractions: *n*-hexane (13.60 g), chloroform (8.40 g), and ethyl acetate (3.10 g). For subsequent biological and photometric assays, the dried residues of these fractions—including the non-polar *n*-hexane and chloroform fractions—were completely re-dissolved in an appropriate amphiphilic vehicle (e.g., absolute methanol or DMSO) to guarantee absolute homogeneity and prevent any phase separation upon mixing with polar reagent solutions.

### 3.3. Phytochemical Screening

A preliminary phytochemical screening of the extracts and the solvent fractions was performed according to previously established protocols [51]. To identify major classes of secondary metabolites—including alkaloids, polyphenols, flavonoids, steroids, triterpenoids, and saponins—the extracts and solvent fractions were dissolved in absolute ethanol at a concentration of 2 mg/mL and reacted with specific diagnostic reagents. The procedures and characteristic observations are summarized in Table 7. For the saponin frothing test, 1 mL of the stock solution was diluted with 10 mL of distilled water in a test tube and shaken vigorously, and then allowed to stand for 15 min. A stable foam height of at least 1 cm after 1 min was taken as a positive indication of saponins. The stability of the generated foam was evaluated by independently adding a few drops of 0.1 N HCl and 0.1 N NaOH. Other screening tests for alkaloids, flavonoids, polyphenols, and triterpenoids were performed by reacting the sample with their respective diagnostic reagents under optimized ethanolic conditions.

### 3.4. Quantification of Total Bioactive Contents

#### 3.4.1. Total Polyphenol Content

The total polyphenol content (TPC) was determined using the Folin–Ciocalteu assay as described previously [52] with minor modifications. Briefly, a reaction mixture containing 250 µL of the extract or solvent fraction, 250 µL of distilled water, and 250 µL of Folin–Ciocalteu reagent was thoroughly vortexed. Subsequently, 250 µL of 10% Na_2_CO_3_ was added, and the mixture was incubated at 40 °C for 30 min. The absorbance of the resulting solution was measured at 765 nm using a microplate reader (SH-1200, Corona Electric, Ibaraki, Japan). The total polyphenol content was calculated based on a gallic acid standard curve and expressed as mg gallic acid equivalents (GAE) per gram of sample.

#### 3.4.2. Total Flavonoid Content

The total flavonoid content (TFC) was quantified according to the aluminum chloride colorimetric method with slight modifications [53]. A 200 µL aliquot of the extract or solvent fraction was mixed with 200 µL of distilled water and 40 µL of 5% NaNO_2_, followed by a 5 min incubation. Then, 40 µL of 10% AlCl_3_ was added to the mixture. After incubating for an additional 6 min, 400 µL of 1 M NaOH was added, and the total volume was adjusted to 1 mL with distilled water. The absorbance was measured at 510 nm using a microplate reader (SH-1200, Corona Electric, Ibaraki, Japan). The total flavonoid content was determined using a quercetin standard curve and expressed as mg quercetin equivalents (QE) per gram of sample.

#### 3.4.3. Total Triterpenoid Content

The total triterpenoid content (TTC) was determined by a colorimetric assay as previously described [54], with minor modifications. Briefly, 10 mg of the extract or solvent fraction was dissolved in 1 mL of methanol. A 100 µL aliquot of each sample solution was subsequently mixed with 150 µL of a vanillin–acetic acid solution (5% *w*/*v* vanillin in glacial acetic acid) and 500 µL of perchloric acid. The reaction mixtures were heated at 60 °C for 45 min and then immediately cooled in an ice-water bath to ambient temperature. After the addition of 2.25 mL of glacial acetic acid, the absorbance of each solution was measured at 548 nm using a microplate reader (SH-1200, Corona Electric, Ibaraki, Japan). Oleanolic acid was employed as the standard, and the results were expressed as mg oleanolic acid equivalents (OAE) per gram of sample.

### 3.5. In Vitro Antioxidant Activity Assays

To establish a mechanistic rationale for the multitarget biological screening, the in vitro antioxidant assays were systematically conducted first. The resulting radical scavenging capacities were then utilized as the baseline biochemical foundation to evaluate the subsequent antidiabetic (α-amylase and β-cell), anticancer, and anti-inflammatory properties, as oxidative stress mitigation is intrinsically linked to these diverse cellular protective functions.

#### 3.5.1. DPPH Radical Scavenging Assay

The DPPH (1,1-Diphenyl-2-picrylhydrazyl) radical scavenging activity of the extract or solvent fraction was determined according to the method of Silva et al. (2024) with minor modifications [55]. Briefly, 100 µL of the extract or solvent fraction at various concentrations (12.50, 25, 50, 100, 200, 400, and 800 µg/mL in ethanol) was added to 96-well plates containing 100 µL of DPPH solution (1 mg/mL in ethanol). The reaction mixture was incubated in the dark for 60 min at room temperature, and the absorbance was subsequently measured at 517 nm using a microplate reader (SH-1200, Corona Electric, Ibaraki, Japan). The antioxidant capacity was expressed as the IC_50_ value (the concentration required to scavenge 50% of DPPH radicals), calculated from the linear regression equation of the scavenging percentage versus sample concentration. Ascorbic acid served as the positive control. All experiments were performed in triplicate.

#### 3.5.2. ABTS Radical Scavenging Assay

The ABTS+ radical scavenging capacity was evaluated following the method described by Platzer et al. (2021) with slight adjustments [56]. Aliquots of 10 µL of the extract or solvent fraction (6.25, 12.50, 25, 50, 100, 200, and 400 µg/mL) were mixed with 990 µL of the ABTS+ solution and incubated for 6 min at room temperature. The absorbance was then measured at 734 nm using a microplate reader (SH-1200, Corona Electric, Ibaraki, Japan). The antioxidant potential was determined based on the IC_50_ value, with ascorbic acid used as the positive control. Each experiment was conducted in triplicate.

#### 3.5.3. Reducing Power (RP) Assay

The iron-reducing capacity was determined using the method of Ouahhoud et al. (2022) with minor modifications [57]. A 500 µL aliquot of the extract or solvent fraction (6.25, 12.50, 25, 50, 100, 200, and 400 µg/mL) was mixed with 500 µL of phosphate buffer (0.2 M, pH 6.8) and 500 µL of 1% K_3_Fe(CN)_6_. The mixture was incubated at 50 °C for 20 min. Subsequently, 500 µL of 10% trichloroacetic acid was added, and the mixture was centrifuged at 3000 rpm for 10 min. A 500 µL portion of the supernatant was collected and mixed with 500 µL of distilled water and 100 µL of 0.1% FeCl_3_. The absorbance of the resulting solution was measured at 700 nm using a microplate reader (SH-1200, Corona Electric, Ibaraki, Japan). The reducing power was expressed as the Abs_0.5_ value—the concentration at which the absorbance reached 0.5—calculated from the plot of absorbance versus concentration. Gallic acid was used as the positive control, and all assays were performed in triplicate.

### 3.6. In Vitro α-Amylase Inhibition and Pancreatic β-Cell Cytoprotective Assays

#### 3.6.1. α-Amylase Inhibitory Activity

The α-amylase inhibitory activity of the extracts was evaluated according to the method of Trang et al. with minor modifications [58]. Briefly, a mixture consisting of 50 μL of phosphate buffer (pH 7.0), 50 μL of the extract or solvent fractions, and 50 μL of α-amylase enzyme (from *Bacillus subtilis*; 3 U/mL) was incubated at 37 °C for 5 min. Subsequently, 50 μL of starch solution (soluble, 2 mg/mL) was added, and the incubation was continued at 37 °C for an additional 15 min. The reaction was terminated by the addition of 200 μL of concentrated HCl. Finally, 300 µL of iodine reagent (1%) was added, and the absorbance was measured at 660 nm using a microplate reader (SH-1200, Corona Electric, Ibaraki, Japan). Acarbose served as the positive control. The IC_50_ value was calculated from the plot of inhibition percentage versus sample concentration.

#### 3.6.2. Cytotoxicity Assay

Cell viability was determined using the CCK-8 (Cell Counting Kit-8) assay. Stock solutions of the samples were prepared in DMSO and diluted with fresh culture medium so that the final DMSO concentration was fixed at 0.1% (*v*/*v*) across all sample concentrations. Briefly, β-TC6 cells were seeded in 96-well plates at a density of 1 × 10^4^ cells/well and cultured for 24 h at 37 °C under a 5% CO_2_ atmosphere. The culture medium was then removed, and the cells were washed with PBS. After adding fresh medium containing the extract or solvent fraction at various concentrations, the plates were incubated for an additional 24 h. Subsequently, the medium was replaced with medium containing CCK-8 solution (10:1, *v*/*v*). The absorbance was measured at 450 nm at 0 and 3 h after the addition of the CCK-8 reagent. Cell viability was expressed as a percentage relative to the untreated control group (medium containing 0.1% DMSO). To evaluate the potential influence of residual solvents on cell viability, a control assay was performed using pure solvents (methanol, *n*-hexane, and chloroform) at the equivalent concentrations used in the final tests. The results confirmed that these controls exerted no significant cytotoxic effects, verifying that the observed bioactivities were solely attributable to the plant metabolites.

#### 3.6.3. Protective Effects Against Thapsigargin

The capacity of the extracts to protect cells from thapsigargin -induced cell death was evaluated. β-TC6 cells were seeded in 96-well plates at a density of 2 × 10^4^ cells/well. After 24 h of incubation at 37 °C and 5% CO_2_, the medium was removed, and the cells were washed with PBS. The cells were then co-treated with medium containing different concentrations of the extract or solvent fraction and thapsigargin (TG; final concentration 2 μM), a potent inducer of ER stress. After 24 h of incubation, the medium was replaced with fresh medium containing CCK-8 solution (10:1, *v*/*v*). Absorbance was recorded at 450 nm at 0 and 3 h post-CCK-8 addition. The cytoprotective effect was determined by comparing the cell viability of extract-treated cells to that of the TG-only-treated control.

### 3.7. Anti-Inflammatory Assay

#### 3.7.1. Cell Viability Assay on RAW 264.7 Cells

RAW 264.7 cells (4 × 10^4^ cells/well) were cultured in a 96-well plate for 24 h [59]. Subsequently, the cells were washed with PBS buffer and treated with serial concentrations of the extract or solvent fraction for 24 h, followed by incubation with or without 1 μg/mL lipopolysaccharide (LPS; from *Escherichia coli*, Sigma-Aldrich, St. Louis, MO, USA) for 18 h. After removing the supernatant, the culture medium was replaced with fresh medium containing a 10:1 (*v*/*v*) mixture of CCK-8 solution in each well and incubated for another 3 h. The absorbance at 450 nm was measured using a microplate reader (SH-1200; Corona Electric, Ibaraki, Japan).

#### 3.7.2. NO Production Inhibitory Assay

RAW 264.7 cells (6 × 10^5^ cells/well) were cultured in a 24-well plate for 24 h. Subsequently, the cells were washed with PBS buffer and pre-treated with serial concentrations of the extract or solvent fraction for 24 h. After replacing the culture medium, the cells were co-treated with extract or solvent fraction in the presence or absence of 1 μg/mL LPS (from *Escherichia coli,* Sigma-Aldrich, St. Louis, MO, USA) for an additional 24 h. To quantify nitric oxide (NO), 100 μL of the culture supernatant was mixed with an equal volume of Griess reagent (1%) in a 96-well plate, and the plate was incubated for 15 min at room temperature [59]. The absorbance of the reaction mixture was measured at 540 nm with an SH-1200 microplate reader (Corona Electric, Ibaraki, Japan). *N*-Nitro-*L*-arginine methyl ester (L-NAME) (100 μM) was used as a standard iNOS inhibitor (positive control).

### 3.8. Anticancer Assay

In this study, cancer cell lines including HeLa (cervical cancer), HT-29 (human colorectal cancer), HepG2 (liver cancer), and A549 (lung cancer), along with HEK293 (normal kidney cells), were used for evaluating the cytotoxicity of the leaf samples using the CCK-8 assay [60]. Briefly, cells (4 × 10^4^ cells/well) were cultured in a 96-well plate for 24 h. Subsequently, the cells were washed with PBS buffer and treated with serial concentrations of the extracts or solvent fractions for 48 h. After removing the supernatant, the culture medium was replaced with fresh medium containing a 10:1 (*v*/*v*) mixture of CCK-8 solution in each well and incubated for an additional 3 h. The absorbance at 450 nm was measured using a microplate reader SH-1200 (Corona Electric, Ibaraki, Japan). The half-maximal inhibitory concentration (IC_50_) value was determined using GraphPad Prism 9 software.

### 3.9. Chemical Composition Analysis

The chemical compositions of the *n*-hexane fractions were determined at the Can Tho Technical Center for Standards, Metrology, and Quality (Can Tho city, Vietnam) using a Gas Chromatography–Mass Spectrometry (GC-MS, GCMS-QP2020 NX, Shimadzu Corp., Kyoto, Japan) system coupled with an electron ionization (EI) source. The analysis was conducted using a TG-SQC column (15 m × 0.25 mm × 0.25 µm) with helium as the carrier gas. The analytical conditions were as follows: electron ionization (EI) source temperature of 230 °C, transfer line temperature of 275 °C, and mass range of *m*/*z* 40–500. The injection volume was 1 μL, utilizing a split injection mode with a split ratio of 1:40 and a split flow of 50 mL/min. The carrier gas flow rate was 1.2 mL/min. The oven temperature program was set as follows: initial temperature at 60 °C (held for 2 min): Ramp 1, increased to 150 °C at a rate of 5 °C/min (held for 1 min); Ramp 2, increased to 200 °C at 10 °C/min (held for 1 min); Ramp 3, increased to 250 °C at 10 °C/min (held for 1 min). The identification of volatile and semi-volatile secondary metabolites was achieved by comparing their mass spectra fragmentation patterns with data stored in the National Institute of Standards and Technology (NIST) (NIST-14, Gaithersburg, MD, USA) and Wiley (Wiley Science Solutions, Hoboken, NJ, USA) mass spectral library, along with the verification of retention indices based on literature data [61].

### 3.10. Molecular Docking and ADMET Prediction

Molecular docking analysis was performed using PyRx 0.8. The crystal structures of the target enzymes, iNOS (PDB: 4NOS) and COX-2 (PDB: 5KIR), were retrieved from the Protein Data Bank (https://www.rcsb.org/). Prior to docking, the proteins were rigorously prepared using Discovery Studio Visualizer software (v25.1.0.0) by systematically removing water molecules, heteroatoms, and native co-crystallized ligands, followed by the addition of polar hydrogens. Ligand structures, alongside the positive controls (celecoxib and indomethacin), were obtained from PubChem (https://pubchem.ncbi.nlm.nih.gov/, accessed on 22 September 2025). To ensure highly accurate and stable thermodynamic conformations, the ligands were geometrically optimized using density functional theory (DFT) at the 6-31G(d) basis set using Gaussian 16 software. To validate the accuracy and reliability of the docking protocol, a redocking validation procedure was performed. The native co-crystallized ligands were extracted from the PDB complexes and re-docked into their respective active sites. The methodology was validated as the Root Mean Square Deviation (RMSD) between the predicted re-docked conformation and the experimental crystallographic pose was confirmed to be ≤ 2.0 Å. Following successful validation, the docking sites for the identified phytoconstituents were defined based on the coordinates of the co-crystallized ligands within each target protein. Specifically, the grid box centers were set at X:Y:Z = 23.7019:93.8031:18.0962 for iNOS and X:Y:Z = 23.2686:1.3399:26.9335 for COX-2.

Pharmacokinetic parameters, including absorption, distribution, metabolism, excretion, and toxicity (ADMET), were predicted using the pkCSM online server. Additionally, the drug-likeness of the potential compounds was evaluated according to Lipinski’s rule of five using an online assessment tool [50,62].

### 3.11. Statistical Analysis

Experimental data were analyzed using GraphPad Prism 9 software (GraphPad Software, San Diego, CA, USA) and Minitab 22.2.0. software (Minitab, LLC, State College, PA, USA). Values are presented as the mean ± standard deviation (SD). Prior to statistical analysis, the normality of the data was assessed and confirmed using the Shapiro–Wilk test. The statistical significance of the differences was determined using Student’s *t*-tests and one-way analysis of variance (ANOVA). A difference was considered statistically significant if the *p*-value was less than 0.05.

## 4. Conclusions

This study provides a comprehensive evaluation of the phytochemical profile and multitarget biological potential of *Crinum asiaticum* L. var. *anomalum* Baker leaves. Our findings demonstrate that the methanol extract, along with the solvent fractions, exhibits diverse biological activities, including antioxidant, anti-inflammatory, and anticancer effects. Furthermore, these extracts showed significant antidiabetic potential by inhibiting α-amylase activity and conferring vital cytoprotection to pancreatic β-cells to support cellular health. Notably, the *n*-hexane, chloroform, and ethyl acetate fractions exhibited divergent biological activities; this variation likely reflects the differing concentrations of key secondary metabolites, such as phenolics, flavonoids, and alkaloids, within each fraction.

Detailed GC-MS analyses of the *n*-hexane fractions allowed for the identification of several bioactive constituents. In silico molecular docking studies suggested that phytol, a major component of the *n*-hexane fraction, is a strong anti-inflammatory candidate due to its high binding affinity for iNOS and COX-2. The in silico ADMET analysis further supports the drug-likeness of these bioactive compounds, revealing favorable pharmacokinetic profiles and high safety margins.

In conclusion, *C. asiaticum* L. var. *anomalum* Baker serves as a highly promising source of bioactive compounds for future functional food and pharmaceutical applications. These findings establish a solid scientific foundation for further investigations focused on the isolation of pure compounds and in vivo studies to fully validate the therapeutic efficacy of this botanical variety.

## Figures and Tables

**Table 1:** The phytochemical composition of leaf methanol extract and its solvent fractions.

Extract/Solvent Fractions	Polyphenols	Flavonoids	Alkaloids	Saponins	Triterpenoids	Glycosides
Methanol extract	+	+	+	+	+	+
*n*-Hexane fraction	+	+	+	+	+	+
Chloroform fraction	+	+	+	+	+	+
Ethyl acetate fraction	+	+	−	+	+	+

Note: (−) absent; (+) present.

**Table 2:** Total polyphenol, total flavonoid, and total triterpenoid contents of the extracts and the solvent fractions derived from the methanol extract of CAVA leaves.

Extract/Fractions	TPC (mg GAE/g Extract)	TFC (mg QE/g Extract)	TTC (mg OAE/g Extract)
Methanol extract	59.55 ± 1.12 ^c^	391.94 ± 5.15 ^a^	223.50 ± 8.66 ^c^
*n*-Hexane fraction	40.06 ± 1.95 ^d^	171.94 ± 1.57 ^d^	740.17 ± 6.01 ^a^
Chloroform fraction	71.02 ± 0.99 ^b^	223.61 ± 2.83 ^b^	502.94 ± 8.22 ^b^
Ethyl acetate fraction	111.71 ± 1.63 ^a^	202.50 ± 2.36 ^c^	151.83 ±8.82 ^d^

Results are expressed as mean ± SD (*n* = 3). TPC: total polyphenol content; TFC: total flavonoid content; TTC: total triterpenoid content; GAE: gallic acid equivalents; QE: quercetin equivalents; OAE: oleanolic acid equivalents. Different superscript letters (a, b, c, d) within the same column indicate statistically significant differences among groups based on One-way ANOVA followed by Tukey’s multiple comparison test (*p* < 0.05).

**Table 3:** Antioxidant effects of the methanol extract and the solvent fractions derived from the methanol extract of CAVA leaves.

Extracts/Fractions	DPPH Radical Scavenging Activity (IC_50_, µg/mL)	ABTS+ Scavenging Activity (IC_50_, µg/mL)	Reducing Power (Abs_0.5_, µg/mL)
Methanol extract	415.00 ± 1.18 ^b^	33.23 ± 1.64 ^b^	165.81 ± 3.65 ^b^
*n*-Hexane fraction	538.90 ± 3.57 ^a^	82.36 ± 3.94 ^a^	306.85 ± 4.78 ^a^
Chloroform fraction	306.70 ± 1.56 ^c^	20.41 ± 5.48 ^c^	132.03 ± 9.15 ^c^
Ethyl acetate fraction	120.00 ± 1.78 ^d^	18.25 ± 3.65 ^c^	67.07 ± 2.28 ^d^
Ascorbic acid	9.70 ± 1.34 ^e^	4.64 ± 1.05 ^d^	–
Gallic acid	–	–	1.57 ± 0.12 ^e^

Results are expressed as mean ± SD (*n* = 3). Ascorbic acid and gallic acid were used as reference control. –, not determined. Different superscript letters (a, b, c, d and e) within the same column indicate statistically significant differences among groups based on one-way ANOVA followed by Tukey’s multiple comparison test (*p* < 0.05).

**Table 4:** IC_50_ values and selectivity index (SI) of the CAVA leaf extract and its sub-fractions against various cancer cell lines. SI values were calculated as the ratio of the IC_50_ value for HEK293 cells to that for each respective cancer cell line.

Extracts	IC_50_/SI	HeLa Cells	HT-29 Cells	HUH-7 Cells	HepG2 Cells	A549 Cells
Methanol extract	IC_50_ (µg/mL)	17.35 ± 1.30 ^c^	10.84 ± 3.25 ^d^	26.92 ± 1.57 ^b^	24.62 ± 7.50 ^b^	44.14 ± 9.57 ^b^
	SI	1.34	2.14	0.86	0.94	0.53
*n*-Hexane fraction	IC_50_ (µg/mL)	65.12 ± 3.45 ^a^	47.30 ± 4.35 ^b^	53.18 ± 0.40 ^a^	110.80 ± 9.47 ^a^	71.02 ± 7.15 ^a^
	SI	1.35	1.85	1.65	0.79	1.23
Chloroform fraction	IC_50_ (µg/mL)	7.10 ± 0.56 ^d^	33.21 ± 4.44 ^c^	7.24 ± 1.39 ^d^	10.69 ± 1.73 ^b^	24.53 ± 2.78 ^c^
	SI	4.34	0.93	4.26	2.88	1.26
Ethyl acetate fraction	IC_50_ (µg/mL)	26.87 ± 1.21 ^b^	87.96 ± 7.72 ^a^	14.63 ± 1.84 ^c^	13.78 ± 2.02 ^b^	35.47 ± 6.61 ^b,c^
	SI	1.40	0.43	2.57	2.73	1.06

Data are expressed as mean ± SD (*n* = 3). Different superscript letters (a, b, c, d) within the same column indicate statistically significant differences among groups based on One-way ANOVA followed by Tukey’s multiple comparison test (*p* < 0.05).

**Table 5:** Lipinski’s rule of five and physicochemical properties of the major constituents (H1–H6) identified in the *n*-hexane fraction of CAVA leaf methanol extract.

Drug-Likeness Criteria		H1	H2	H3	H4	H5	H6
Molecular weight	<500 Da	282.468	296.539	164.204	368.646	204.357	138.254
Hydrogen bond acceptors	<10	1	1	2	2	0	0
Hydrogen bond donors	<5	1	1	1	0	0	0
Molar refractivity	40–130	89.94	98.94	49.06	118.77	72.32	49.24
Lipophilicity (log *P*)	<5	6.1085	6.3641	2.1293	8.3714	5.2015	3.699

H1: oleic acid; H2: phytol; H3: eugenol; H4: ethyl docosanoate; H5: (*Z*)-β-Farnesene; H6: 2-methyl-6-methylene-2-octene.

**Table 7:** Diagnostic reagents and characteristic observations for qualitative phytochemical screening.

Chemical Constituents	Test/Reagent	Observation and Inference
Polyphenols	10% FeCl_3_ solution	Development of a characteristic dark green or blue-black coloration
Flavonoids	Concentrated H_2_SO_4_	Development of deep yellow to orange colors (flavones/flavanols); deep red to blue-red (chalcones/aurones); or orange to red (flavanones).
Alkaloids	Dragendorff’s reagent	Formation of a distinct orange or orange-red precipitate.
Saponins	Frothing test (evaluated with HCl 0.1 N and NaOH 0.1 N)	Formation of a persistent and durable foam (honeycomb-like frothing).
Triterpenoids	Liebermann–Burchard test	Formation of a red, pink, or violet ring at the interface.
Glycosides	Keller–Kiliani test	Formation of a reddish-brown ring at the interface between the two liquid layers.

## Data Availability

All data generated or analyzed during this study are included in this published article and its Supplementary Materials. Raw datasets supporting the reported results are available from the corresponding author upon reasonable request.

## Supplementary Materials

The following supporting information can be downloaded at: https://www.mdpi.com/article/10.3390/plants15131957/s1, Figure S1: GC-MS chromatogram of *n*-hexane fraction derived from *C. asiaticum* leaf methanol extract; Table S1: Molecular docking affinities with ligands and target enzymes.

## Abbreviations

The following abbreviations are used in this manuscript:

ABTS	2,2-azino-bis-3-ethylbenzothiazoline-6-sulphonic acid
DPPH	1,1-Diphenyl-2-picrylhydrazyl
TPC	Total polyphenol content
TFC	Total flavonoid content
TTC	Total triterpenoid content
RP	Reducing Power

## Appendix A

**Table A1:** The chemical composition of *C. asiaticum* L. var. *anomalum* Baker *n*-hexane fraction.

Peak	Retention Time (t_R_, min)	Area	% Area (%)	Height	Name
1	6.409	55,834	0.34	28,528	6-Methyl-5-heptene-2-one
2	7.582	210,621	1.30	84,597	2-Methyl 6-methylene 2-octene
3	7.695	33,011	0.20	20,090	3,7-Dimethyldecane
4	8.362	30,729	0.19	18,059	Dodecane
5	9.896	65,815	0.41	32,426	6-Methylheptane-1,6-diol
6	10.065	29,498	0.18	19,816	Cryptone
7	11.176	69,175	0.43	41,793	Hexadecane
8	11.456	80,606	0.50	42,098	*p*-Propenylphenyl methyl ether
9	11.880	51,011	0.31	26,997	6-Propyltridecane
10	12.329	62,299	0.38	33,695	2-Bromomethyl-3,4,5,6-tetramethoxytetrahydropyran
11	12.420	378,753	2.33	177,979	Eugenol
12	13.725	71,490	0.44	39,860	*cis*-Geranylacetone
13	14.256	59,290	0.37	28,227	Hexadecane
14	14.809	238,167	1.47	100,244	(*Z*)-β-Farnesene
15	14.887	257,957	1.59	59,323	Ethyl docosanoate
16	15.053	103,426	0.64	42,571	(2,6,6-Trimethyl-2-hydroxycyclohexylidene) acetic acid lactone
17	15.165	102,049	0.63	43,006	Dodecanoic acid
18	15.379	938,382	5.78	195,286	Phytol
19	15.944	13,395,775	82.52	3,438,531	Oleic Acid
Total		16,233,888	100.00	4,473,126	
