# Supplementary material for: Phytochemical Profiling and Multitargeted Biological Activities of Crinum asiaticum L. var. anomalum Baker Leaf: In Vitro and In Silico Insights
Source: Plants (Basel). 2026 Jun 25;15(13):1957. doi: 10.3390/plants15131957 (PMC13364001; doi:10.3390/plants15131957)
Supplement: Supplementary file 1 [file plants-15-01957-s001.zip › plants-4386103-supplementary.pdf]

Article

Phytochemical Profiling and Multitargeted Biological Activities of *Crinum asiaticum* L. var. *anomalum* Baker Leaf: *In Vitro* and *In Silico* Insights

Tue Minh Duong[1], Son Hoang Nguyen[1,2], Kiep Minh Do[2], Tran Thanh Men[3], Kenji Kanaori[1,*] and Kaeko Kamei[1,*]

[1] Department Functional Chemistry, Kyoto Institute of Technology, Kyoto 606-8585, Japan
[2] Faculty of Biochemistry and Food Technology, Vinh Long University of Technology Education, Vinh Long 85000, Vietnam
[3] Faculty of Biology, College of Natural Science, Can Tho University, Can Tho 94000, Vietnam
* Correspondence: kanaori@kit.ac.jp (K.K.); kame@kit.ac.jp (K.K.); Tel./Fax: +81-75-724-7825 (Kenji Kanaori)

Supplemetary materials

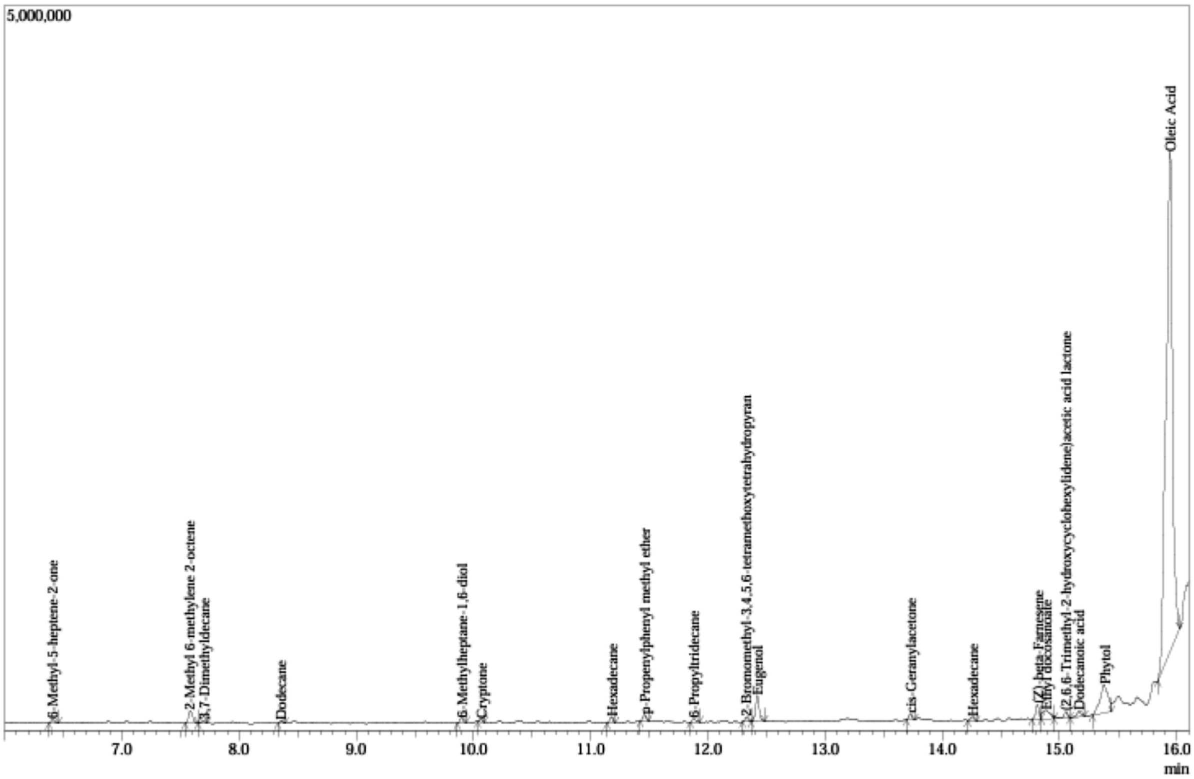

Figure S1. GC-MS chromatogram of *n*-hexane fraction derived from *C. asiaticum* leaf methanol extract

Table S1. Molecular docking affinities with ligands and target enzymes

Compounds	*i*NOS (PDB: 4NOS)			COX-2 (PDB: 5KIR)		
	Binding affinity (kcal/mol)	Interaction bonds		Binding affinity (kcal/mol)	Interaction bonds	
		Conventional hydrogen/Carbon hydrogen/Alkyl/Pi-alkyl/Pi-Pi Stacked/Pi-Pi T shaped/Pi-Sigma/Unfavorable Aceptor-Aceptor/Pi-Lone pair/Halogen (Flourine)	van der Waals		Conventional hydrogen/Carbon hydrogen/Alkyl/Pi-alkyl/Pi-Pi Stacked/Pi-Pi T shaped/Pi-Sigma/Unfavorable Aceptor-Aceptor/Pi-Lone pair/Halogen (Flourine)	van der Waals
Celecoxib	-10	Glu377, Trp372, Gly202, Pro350, Tyr489, Cys200, Phe369, Gly371, Trp194	Tyr373, Gln263, Ala351, Val352, Asn370, Gln205, Ser242	-11.4	Gln192, Arg513, Ser353, Phe518, Leu352, Val349, Val116, Val523, Tyr355, Leu531, Leu359, Ala527, Leu384, Tyr385, Trp387	Arg120, His90, Gly354, Ala516, Ile517, Ser530, Gly526, Met522
Indomethacin	-8.9	Trp194, Phe369, Cys200, Val352, Pro350, Tyr489, Ile244, Leu209	Gly202, Ile201, Met374, Glu377, Trp372, Gln263, Ala351, Asn370, Gly371, Ser242	-7.4	Arg120, Glu524, Lys83, Tyr115, Val116, Leu93, Tyr365, Ile92, Val89, Trp100	Pro84, Pro86
Oleic acid	-7.1	Tyr491, Leu125, Phe488, Arg199, Ala197, Phe369, Tyr489, Cys200, Leu209, Trp194	Met355, Pro198, Asn370, Gly371, Pro360	-6.7	Val523, Phe518, Ala516, Trp387, Leu352, Ala527, Val349, Leu531, Tyr355, Arg120	Gln192, Ile517, His90, Arg513, Leu359, Val116, Tyr348, Ser530, Tyr385,

						Gly526, Met522, Ser353
Phytol	-7.4	Arg199, Tyr489, Ala197, Phe369, Cys200, Leu209	Pro198, Phe488, Tyr491, Met355, Asn370, Gly371	-7.2	Val523, Leu352, Ala527, Val349, Tyr385, Tyr348, Phe209, Leu534, Phe381, Phe205	Gly227, Asn375, Val223, Ile377, Gly533, Ser530, Trp387, Gly525, Phe518, Ser353
Eugenol	-7.3	Phe369, Trp194, Cys200	Tyr489, Leu209, Ala243, Asn370, Ser242, Gly371	-6.6	Tyr206, His388, His207, Ala202, Leu391, Phe200, Ala199	Phe210, His386, Gln203, Leu390, Trp387
Ethyl docosanoate	-6.8	Gly371, Trp194, Phe369, Tyr489, Met355, Cys200, Ala197, Arg199, Pro198, Phe488	Asn370, Pro350, Trp372, Glu377, Tyr491	-6.3	Ala527, Val523, Leu352, Val349, Tyr355, Leu359, Leu531, Val116, Leu93, Val89, Tyr115, Ile112	
Z-beta-Farnesene	-7.2	Cys200, Trp194, Phe369, Ala197, Leu209, Tyr489	Gly371, Asn370	-6.8	Val523, Ala527, Leu359, Val116, Leu531, Tyr355, Val349, Leu352, Trp387, Phe518	Gly526, Ser530, Ser353
2-methyl-6-methylene-2-octene	-6.7	Met355, Tyr489, Ala197, Phe369, Trp194, Leu209	-	-5.8	Tyr385, Leu384, Ala527, Trp387, Phe381, Phe518, Leu362, Val523	Met522, Gly526, Val349, Ser363, Ser530
(2,6,6-trimethyl-2-hydroxycyclohexylidene)acetic acid lactone	-6.2	Ala197, Met355, Arg199, Tyr491	Tyr489, Pro198, Trp463, Cys200, Phe369	-6.5	Val349, Leu352, Tyr385	Ser353, Phe518, Ala527, Val523, Met522, Gly526, Trp387,

						Tyr348, Ser530
Dodecanoic acid	-6.1	Leu209, Ala197, Val352, Cys200, Tyr489, Phe369, Trp194, Gly371, Asn370	Ser242, Ala351, Pro350	-5.9	Ala527, Val349, Ala516, Leu352, His90, Val523, Ser530	Gln192, Ile517, Arg513, Ser353, Phe518, Gly526, Leu384, Phe381, Trp387, Tyr348, Tyr385
